# Glycosylation, an effective synthetic strategy to improve the bioavailability of therapeutic peptides

**DOI:** 10.1039/c5sc04392a

**Published:** 2016-01-29

**Authors:** Shayli Varasteh Moradi, Waleed M. Hussein, Pegah Varamini, Pavla Simerska, Istvan Toth

**Affiliations:** a The University of Queensland , School of Chemistry and Molecular Biosciences , Brisbane , QLD 4072 , Australia . Email: i.toth@uq.edu.au; b Institute for Molecular Bioscience , The University of Queensland , St. Lucia , QLD 4072 , Australia; c The University of Queensland , School of Pharmacy , Brisbane , QLD 4072 , Australia

## Abstract

Glycosylation of peptides is a promising strategy for modulating the physicochemical properties of peptide drugs and for improving their absorption through biological membranes. This review highlights various methods for the synthesis of glycoconjugates and recent progress in the development of glycosylated peptide therapeutics. Furthermore, the impacts of glycosylation in overcoming the existing barriers that restrict oral and brain delivery of peptides are described herein.

## Abstract 2

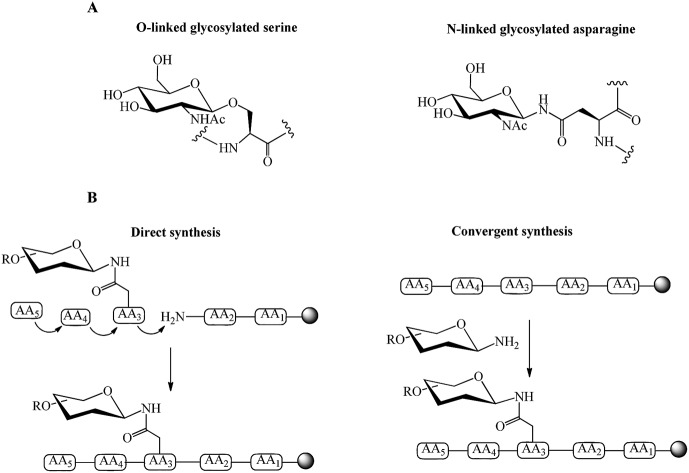
Glycosylation of peptides is a promising strategy for modulating the physicochemical properties of peptide drugs and for improving their absorption through biological membranes.

## 1. Introduction

Peptides have promising therapeutic potential in the treatment of several diseases as they show high activity, target specificity, low toxicity, and minimal non-specific and drug–drug interactions.^1^–^3^ Numerous attempts have been made to improve the pharmacological properties of peptide drugs and deliver them efficiently to the target sites, particularly through non-parenteral routes.^4^–^6^ However, the poor physicochemical properties of peptides impede their efficient delivery. More importantly, oral peptide delivery can be challenging due to biological hurdles, such as variable pH across the gastrointestinal tract (GIT), the presence of proteases and physical barriers.^7^,^8^ For example, the phospholipid bilayer in biological membranes limits the adequate penetration of peptide drugs inside the intestinal cells. Furthermore, inadequate absorption and rapid degradation by proteolytic enzymes are additional obstacles that result in the low oral bioavailability of peptides (less than 1–2%).^8^–^10^

Different strategies have been explored to overcome these obstacles and can be classified into two major groups: (1) chemical modification of peptides, and (2) formulation of peptides (including use of absorption enhancers).^2^,^11^ Glycosylation, PEGylation, lipidation, and cyclisation are examples of chemical approaches to improve the pharmacological profile of the therapeutic peptides.^12^–^14^ Chemical modifications, including the attachment of glycosyl units to peptides, can cause several changes in their features, including their conformational structures and their chemical, physical, and biochemical properties as well as their functions.^15^,^16^

This review describes the barriers that peptide drugs need to overcome, the impact of glycosylation as an effective strategy for peptide delivery and its applications in the development of therapeutic peptides. It also provides insight into synthetic methods for glycoconjugate production.

## 2. Glycosylation strategy for peptide delivery

The introduction of carbohydrate moieties changes the physiological properties of peptides, which can improve their bioavailability. Some advantages of peptide glycosylation can include: (1) targeting specific organs and enhancing biodistribution in tissues,^17^ (2) improving penetration through biological membranes,^18^ (3) increasing metabolic stability and lowering the clearance rate,^19^ (4) receptor-binding,^20^ (5) protecting amino acid's side chain from oxidation,^21^ and (6) maintaining and stabilising the physical properties of peptides, such as precipitation, aggregation and thermal and kinetic denaturation.^4^,^22^,^23^ Conjugation of sugars with peptides can also facilitate the active transport of modified compounds across cell membranes by targeting glucose transporters on the surface of biological membranes.^24^ The favourable impact of glycosylation on pharmacokinetic properties of the native peptides leads to an increase in their oral absorption and bioavailability. Glycosylated somatostatin is one pioneering example with potent oral activity. The oral bioavailability of the modified peptide improved markedly compared to the parent peptide, which resulted in an enhanced inhibitory effect in the release of growth hormone after oral administration.^25^

### 2.1. Strategies for site-specific glycosylation of peptides

#### 2.1.1. N- and O-linked glycosylation

**Fig. 1:**
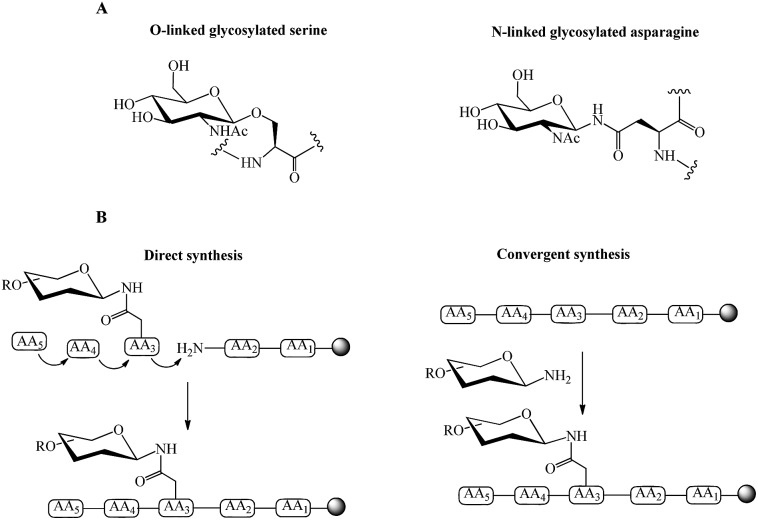
(A) Examples of O-linked and N-linked glycosylated amino acids, (B) direct and convergent strategies for glycopeptide synthesis.

The processes of N- and O-linked glycosylation, in which carbohydrates are attached to polypeptide chains, are naturally occurring. This attachment can be through co-translational or post-translational modifications.^26^ N-linked glycosylation occurs through the amine group of asparagine residue resulting in the formation of an amide bond. In O-linked glycopeptide, the oxygen atom in the side chain of Ser or Thr residues binds to the carbohydrate moiety through an ether bond (Fig. 1A).^26^–^28^ Chemical and chemo-enzymatic methods can be used for the synthesis of glycopeptides and glycoproteins.

Direct and convergent syntheses (Fig. 1B) are two common chemical strategies for the synthesis of N- or O-linked glycopeptides. In the direct method, the pre-synthesised glycosylated amino acid is coupled to the elongating peptide using solid phase peptide synthesis (SPPS) in a stepwise fashion.^28^ Two methods, fluorenylmethyloxycarbonyl (Fmoc) and *tert*-butyloxycarbonyl (Boc) chemistry, are used in SPPS. Generally, glycopeptide synthesis is performed through the Fmoc strategy because the strong acidic condition of Boc-chemistry affects the glycosidic linkages in common oligosaccharide.^29^ The synthesis of long peptides with more than 50 residues is difficult by stepwise synthesis, due to the incomplete couplings and epimerisation. This leads to the formation of side products and a low yield of final product.^30^ Therefore, convergent (fragment-condensation) methods including on-resin linked glycopeptide and Lansbury aspartylation are applied as alternatives to overcome this problem. The convergent approach is particularly used for N-linked glycopeptide synthesis, as O-glycosylation is not achievable by this method.^31^ In these convergent methods, the glycosylamine unit is conjugated to a free Asp residue on a peptide through condensation of the amino acid.^32^ The racemisation of peptide at the C-terminus and formation of aspartimides are the major disadvantages of the convergent methods. Several strategies have been developed to overcome these drawbacks. An on-resin convergent synthesis was reported by Chen and Tolbert in which 2-phenylisopropyl protecting group is used as an orthogonal handle to create glycosylation sites on-resin for the coupling of a large high mannose oligosaccharide to peptides to suppress the aspartamide formation.^33^ Introducing allyl esters and 4-[N-[1-(4,4-dimethyl-2,6-dioxocyclohexylidene)-3-methylbutyl]-amino]benzyl (Dmab) as protecting groups on aspartic acid residues is also an efficient method for selective deprotection and improving the yield of N-linked glycopeptide.^29^ Wang *et al.* reported a modified Lansbury aspartylation for the synthesis of complex glycopeptides. In this method, short glycopeptide fragments were synthesised using convergent aspartylation followed by ligation with a long peptide domain, in which a pseudoproline motif was incorporated into Ser or Thr residues to inhibit the production of aspartimide by-products.^34^

#### 2.1.2. Chemical glycosylation

**Fig. 2:**
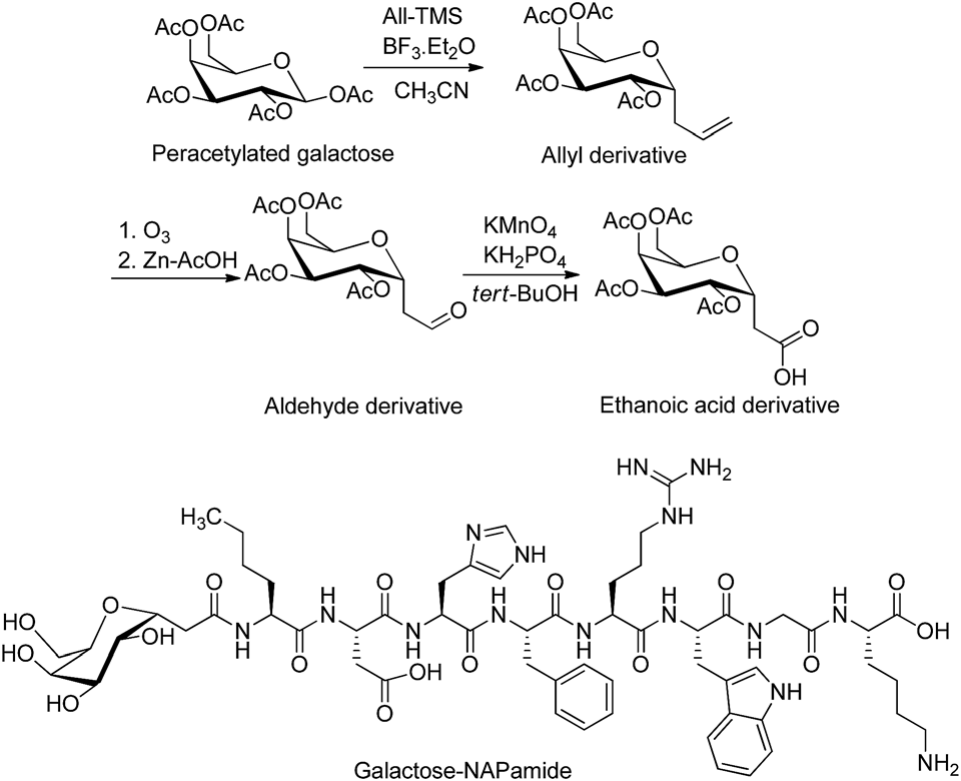
Synthesis of tetra-acetylated galactose ethanoic acid building block *via* the formation of the two intermediates, allyl and aldehyde derivatives. Galactose was conjugated to a peptide through anomeric carbon modified by ethanoic acid.^35^,^37^ The attachment of galactose building block (2 eq.) to the N-terminus of NAPamide peptide on resin was performed on SPPS by using 1-[bis(dimethylamino)methylene]-1H-1,2,3-triazolo[4,5-*b*]pyridinium 3-oxid hexafluorophosphate (HATU) and N,N-diisopropylethylamine (DIPEA) in dimethylformamide (DMF). Removal of acetyl groups from sugar was achieved on solid phase by using hydrazine hydrate/DMF. All-TMS, allyltrimethylsilane; BF_3_·Et_2_O, boron trifluoride diethyl etherate.

**Fig. 3:**
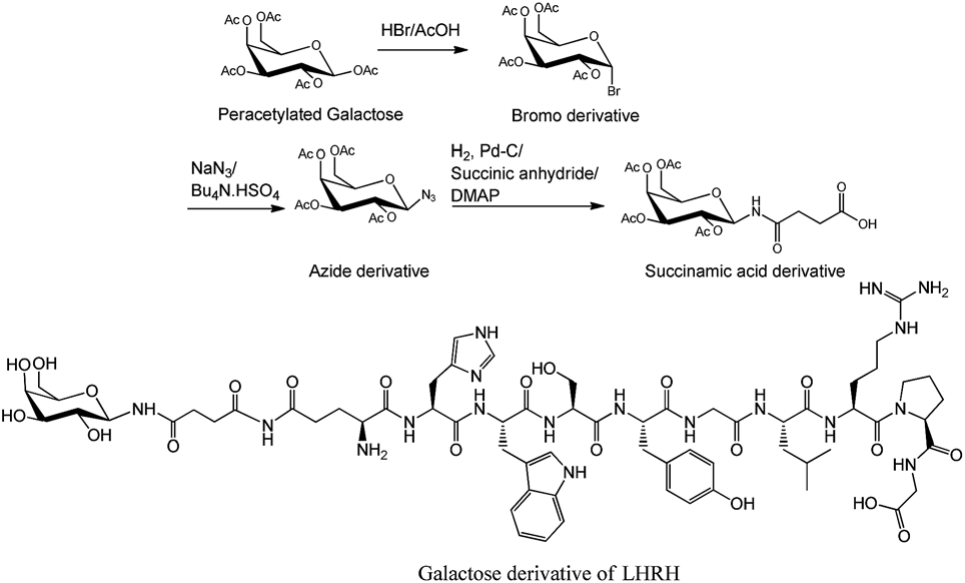
Succinamic acid derivative building block was synthesized through the reduction of azide, followed by treating the resulting product with succinic anhydride.^36^,^38^ Sugar was attached to the peptide through a succinamic acid linker as shown in galactose derivative of luteinizing hormone-releasing hormone (LHRH).^36^ DMAP, 4-dimethylaminopyridine; Pd–C, palladium carbon.

In addition to O- and N-linked glycosylation approaches, several chemical methods have been established for the attachment of carbohydrate units to different amino acid residues at the N-terminus of the peptide's sequence. Conjugation of galactose to the N-terminus of α-melanocyte-stimulating hormone octapeptide analogue (NAPamide) is one of the examples in which the anomeric carbon of the carbohydrate was modified by ethanoic acid and attached to the N-terminus of NAPamide *via* SPPS (Fig. 2).^35^ N-terminus modification of peptides is also achievable by conjugation of carbohydrate units to peptide through a succinamic linker, in which the azide derivative of the sugar moiety is replaced by succinamic acid at the anomeric carbon and coupled to the N-terminus of the peptide through a peptide bond (Fig. 3).^36^

#### 2.1.3. Chemo-enzymatic glycosylation

**Fig. 4:**
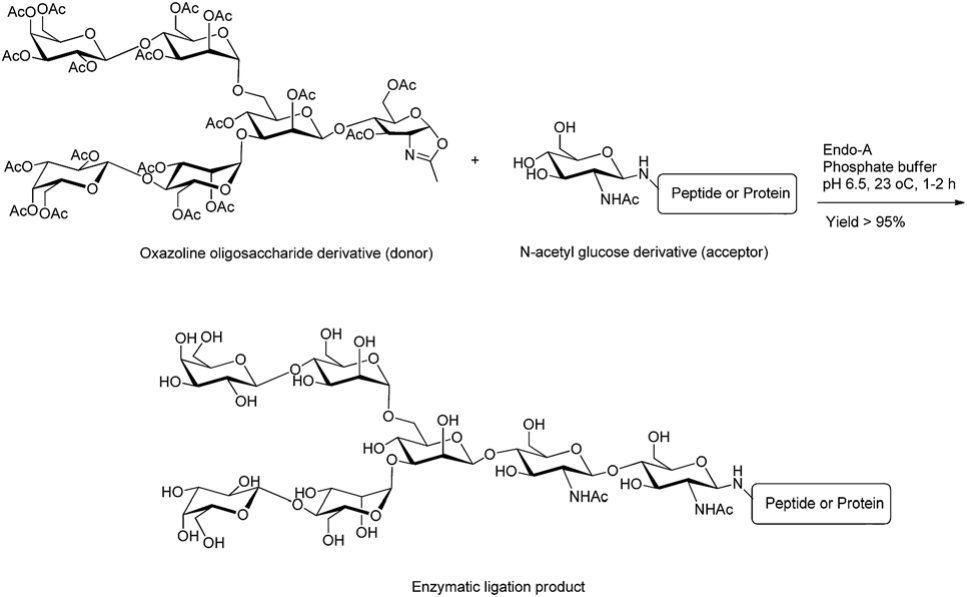
Ligation of oxazoline hexasaccharide (donor) with N-acetyl glucose derivative (acceptor) using Endo-A enzyme.^42^

Chemo-enzymatic approaches are powerful tools that combine the flexibility of chemical synthesis and high regio- and stereo-selectivity of enzyme-catalysed reactions to achieve highly efficient synthesis of complex carbohydrates.^39^,^40^ Particularly, these techniques are ideal choices for complex chemical synthesis, like sialic acid-containing molecules or the attachment of oligosaccharides to polypeptides.^41^*Endo*-β-N-acetylglucosaminidases (ENGases), glycosyltransferases and oligosaccharyltransferases (OST) are the most commonly used enzymes in the chemoenzymatic approach. ENGases are able to couple an intact oligosaccharide to the N-acetylglucosamine (GlcNAc)-containing peptide or protein as an efficient acceptor in a single step.^42^–^44^ In addition to the hydrolysis of the glycosidic bond (cleaving the chitobiose core of N-linked glycans between two GlcNAc residues),^44^ ENGases have transglycosylation activity that can attach the released oligosaccharyl moiety to a suitable acceptor and form a new glycopeptide. Endo-A (from *Arthrobacter*) and Endo-M (from *Mucor hiemalis*) are common ENGases with distinct substrate activity to process oxalines as donors and attach them to GlcNAc derivatives as acceptors (Fig. 4). Endo-A specifically adds high-mannose N-glycans to a variety of acceptors bearing GlcNAc residues, whereas Endo-M acts on the attachment of three major types of N-glycan (high-mannose type, hybrid type, and complex type). Although the transglycosylation activity of each enzymes is unique, their hydrolytic activity results generally in product hydrolysis and restricts their broad application for chemoenzymatic approaches.^45^

**Fig. 5:**
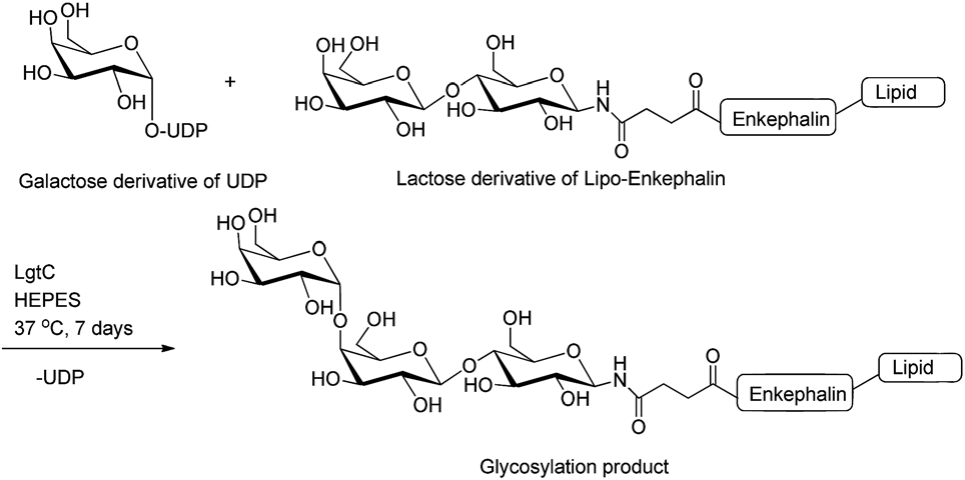
Enzymatic glycosylation of lactose derivative of lipo-enkephalin (acceptor) using UDP-galactose derivative (donor) and LgtC enzyme.^50^ UDP, uridine-5′-diphosphate.

Glycosyltransferases are able to extend the sugar chain by the attachment of one monosaccharyl residue at a time.^45^ β-(1,3)-N-Acetylglucosaminyltransferase (EC 2.4.1.56; LgtA) is an enzyme isolated from *Neisseria meningitides* and was used for the conjugation of GlcNAc residue to the lactose moiety of both endomorphin-1 and enkephalin derivatives.^46^–^48^ Lipopolysaccharyl α-1,4-galactosyltransferase (EC 2.4.1.; LgtC) is another glycosyltransferase derived from *Neisseria meningitides*,^49^ which has been used to attach the galactose unit (Gal) to the terminal lactose residue of lipooligosaccharide.^49^ LgtC was used to attach the Gal residue to a glycosylated enkephalin to improve the metabolic stability of the peptide and target the asialoglycoprotein receptor (ASGPR) (Fig. 5).^50^ The advantages of using glycosyltransferases for glycosyl unit attachment include high regio- and stereo-specificity without the need for protecting groups.

The straightforward separation of the glycopeptide substrates from the glycosyltransferase in the reaction mixture is challenging. It has been shown that the attachment of the polyethylene glycol (PEG) moiety to the N-terminal of Mucin1 (MUC1) through SPPS was an efficient method to facilitate the site-specific enzymatic glycosylation of peptides and the recovery of the final product. In this strategy, the Thr5 residue of N-terminally PEGylated (PEG27 polymer containing 27 oxyethylene units) MUC1 tandem repeat peptide (18 amino acids of the tandem repeat sequence of human MUC1) was glycosylated sequentially in the presence of the recombinant enzyme *Drosophila* glycosyltransferases (dGalNAcT1, dC1GalT1 and dGlcAT-BSII). Glycosyltransferase dGalNAcT1 was employed to specifically attach uridine 5′-diphospho-N-acetylgalactosamine to the Thr5 of the peptide along five possible O-glycosylation sites. The galactose and glucose moieties were coupled to the GalNAc unit at position five using dC1GalT1 and dGlcAT-BSII, respectively, to further elongate the saccharide chain. The glycosylated peptides were then recovered by precipitation and gel-permeation chromatography using a spin column. The presence of monodisperse PEG polymer allowed for quantifiable glycosylation reactions and easy recovery of the glycosylated products without intermediate purification steps.^51^ A more efficient method has been developed recently, in which a photocleavable auxiliary was attached to PEG polymer and cleanly removed by UV irradiation. This auxiliary group improved the efficiency of the enzymatic glycosylation. It also provided the functional group for native chemical ligation to conjugate two or more MUC1 tandem repeats containing a C-terminal thioester moiety. This auxiliary-mediated chemoenzymatic glycosylation approach is applicable to the synthesis of different, larger glycosyl modified proteins. However, it is limited by the identification of a suitable glycine residue, as well as the availability of chemistry or enzymes that introduce the desired glycosylation.^52^

Bacterial OST enzymes are key proteins responsible for N-glycosylation of proteins in bacteria biosynthesis systems. PglB (expressed in *Campylobacter jejuni*) is one these enzymes used in the chemoenzymatic synthesis of glycopeptides/glycoproteins.^53^ In bacterium, this enzyme is involved in N-linked glycosylation of proteins through the transfer of an oligosaccharide from a lipid carrier, undecaprenyl pyrophosphate (Und-PP), to the asparagine side chain of proteins with the consensus sequence D/E-X1-N-X2-S/T, where X1 and X2 can be any amino acids except proline.^54^ The *in vitro* glycosylation activity of PglB has been examined in several studies using synthetic Und-PP glycan as the donor substrate to transfer the oligosaccharyl moieties to a peptide acceptor containing consensus sequence.^53^,^55^,^56^ For instance, mono-, tri- and heptasaccharyl undecaprenyl pyrophosphates were chemically synthesised as donor substrates for PglB OST and conjugated to fluorescent-labelled peptides bearing D/E-X1-N-X2-S/T sequence in the presence of the enzyme. The production of the glycosylated peptides were monitored by sodium dodecyl sulfate polyacrylamide gel electrophoresis (SDS-PAGE) and confirmed by matrix-assisted laser desorption ionization (MALDI) or ESI-TOF MS analysis.^56^

### 2.2. Impact of glycosylation on physicochemical properties of peptides

The physicochemical properties of peptide drugs play an important role in their pharmacokinetic profile and metabolic fate in the human body. Glycosylation can enhance the molecular stability and change the conformation of the peptide backbone.^57^–^59^ Lin *et al.* showed that the modification of hamster prion peptide with different sugar entities, such as mannose, galactose, and N-acetylgalactosamine (GalNAc), exerts diverse impacts on the conformational properties of the polypeptide chain. Mannosylation of the prion exerted an inhibitory impact on the formation of amyloid fibril (a type of aggregation), implying an anti-aggregation function of this sugar entity on the prion peptide.^59^ It has been shown that the position of the glycosyl unit in the peptide's structure is an important factor in changing the conformation of the peptide backbone and may affect the biological properties of the modified peptide. For instance, an attachment of GalNac to Thr^6^ and Thr^21^ in a calcitonin peptide broke the helical structure of the intact peptide, resulting in a reduction in receptor-binding affinity and loss of bioactivity.^60^

### 2.3. Impact of glycosylation on pharmacological characteristics of peptides

**Fig. 6:**
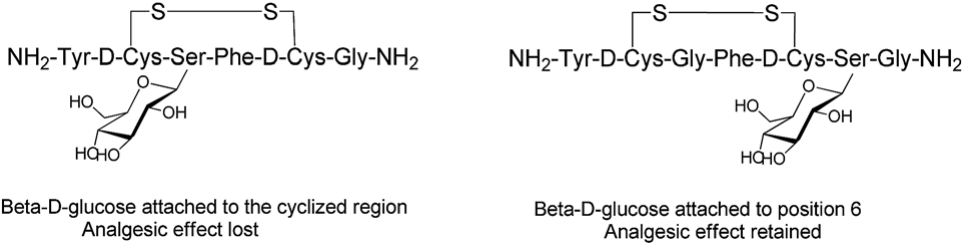
Analogues of Met-enkephalin peptide with β-d-glucose attached to different positions.^64^

Endogenous peptides have typically short half-lives in the biological environment due to enzymatic degradation. Glycosylation can improve the poor pharmacological properties of peptides and the therapeutic efficacy of the formed glycopeptides. Several factors, such as position, type and the number of carbohydrates, are crucial to enhance the pharmacological properties of the manipulated peptides and influence their biological functions.^61^ The position of the glycosyl unit attached to the peptide can influence the peptide–receptor interactions, biodistribution and pharmacological activity of the glycosylated peptides.^35^,^62^,^63^ The structure–activity studies with enkephalin-based glycopeptides demonstrated that the position of the glycosyl units attached to the opioid peptides had different effects on binding affinity and potency of the glycopeptide. The addition of β-d-glucose to the cyclised region of the opioid peptide Met-enkephalin analogue decreased receptor binding and eliminated *in vivo* activity. Whereas, glycosylation at position six of both the cyclised and the linear peptides (Fig. 6) significantly improved analgesic activity after central administration with retained receptor-binding affinity.^64^ If carbohydrate units are attached to peptides at the proper position, they preserve the affinity of native peptide with the target receptor and enable the peptide to be orally active.^35^ The site-dependent effect of glycosylation was also investigated for O-glycosylated calcitonin analogues and it was shown that glycosylation affects both the conformation and biological activity of calcitonin in a site-dependent manner.^65^

The effect of various carbohydrates on renal delivery of vasopressin was studied in rats. The glucosylated and mannosylated vasopressin exhibited higher renal uptake than the galactose-modified analogue that decreased the clearance of the peptide from the body. It was also shown that the glucosyl and mannosyl conjugates were bound specifically to the kidney microsomal membrane *in vitro*, increasing the renal uptake of the peptide.^66^

**Fig. 7:**
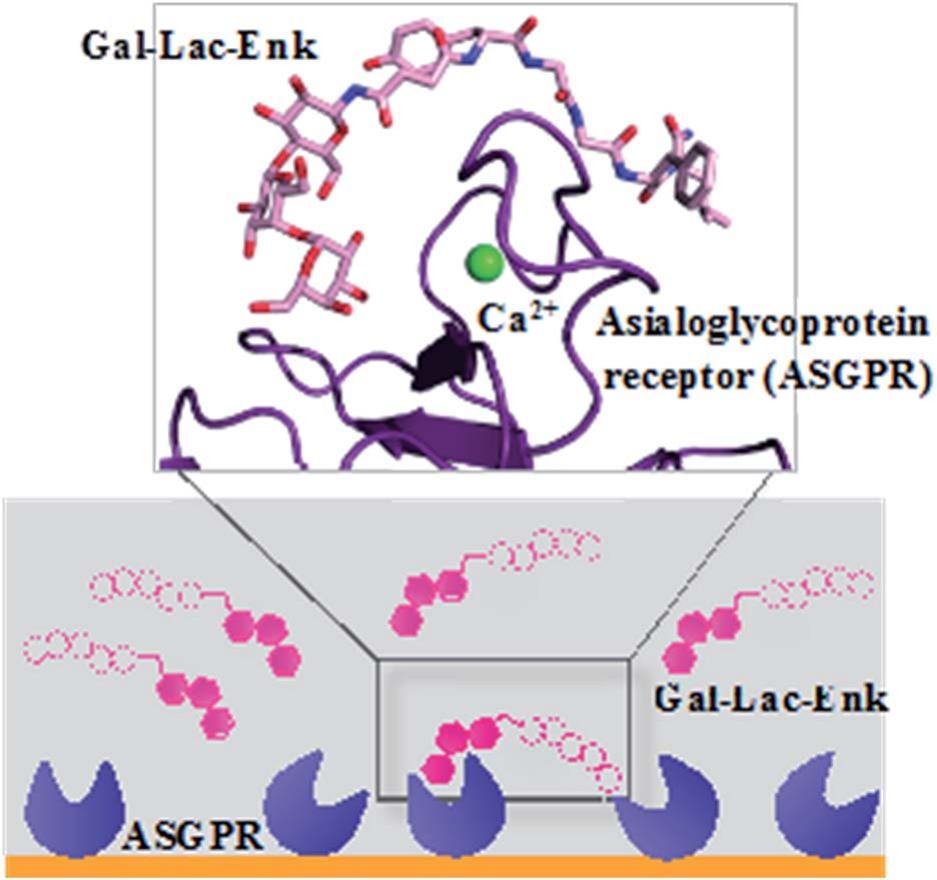
Binding of Gal–Leu–enkephalin to ASGPR displayed by surface plasmon resonance and molecular modelling.^67^ Gal–Lac–Enk, galactose–lactose–enkephalin.

The attachment of the trisaccharide, galactose–lactose, to enkephalin contributed to a 2-fold higher binding affinity of this glycosylated peptide to the ASGPR compared to the binding affinity of the peptide alone (Fig. 7). The enzymatic stability of this trisaccharide–enkephalin improved significantly in human plasma and human colon epithelial cancer cell (Caco-2) homogenates compared to the peptide alone.^67^ However, a higher number of sugar units have not always been accompanied by improved biological properties of the modified peptides. From the tested library of glycopeptides,^65^ a single GlcNAc unit attached to calcitonin had the best hypocalcemic effect with improved biodistribution of the peptide; whereas, increasing the number of carbohydrate moieties (multiple copies of mannose and GlcNAc) decreased the activity of calcitonin.^65^

**Fig. 8:**
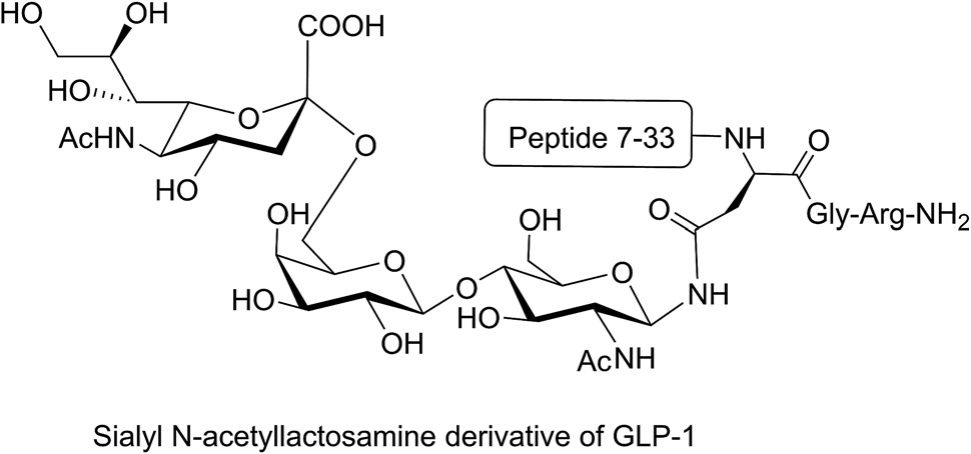
Sialyl *N*-acetyllactosamine derivative of GLP-1. GLP-1 peptide sequence (7–36) was modified by replacing the Lys34 with sialyl N-acetyllactosamine Asn residue. This analogue was synthesised by enzymatic carbohydrate elongation using galactosyltransferase and sialyltransferase.^70^

Different strategies of glycosylation (N-linked, O-linked or N-terminal glycosylation) have been applied for improving the metabolic stability of modified peptides both *in vivo* and *in vitro*. The introduction of *O*-β-glucosylated Ser glucose to the analgesic compound TY027 (Tyr-d-Ala-Gly-Phe-Met-Pro-Leu-Trp-NH-3′,5′-Bzl(CF_3_)_2_) at position six (O-linked glycosylation) enhanced its metabolic stability significantly.^63^ A longer serum half-life was reported for glycosylated major histocompatibility complex (MHC)-binding peptides (MHC receptor inhibitors)^68^ compared to non-glycosylated ones. Substitution of valine with N-acetylglucosamine-modified Asn in MHC-binding peptide (N-linked glycosylation) stabilised the modified peptide against serum peptidases significantly compared to the unmodified analogue.^68^ N-terminal modification of glucagon-like peptide 1 (GLP-1) with glucitol residue improved the resistance of the compound to enzymatic degradation after intraperitoneal administration to Wistar rats.^69^ In another study, the attachment of sialyl N-acetyllactosamine to Asn residue of GLP-1 *via* N-linked glycosylation improved the *in vivo* stability of the modified peptide and prolonged its anti-hyperglycaemic activity (Fig. 8).^70^ N-terminal attachment of the glycosyl unit to endomorphin-1 *via* the succinamic acid linker improved the metabolic stability of the peptide in human serum significantly.^18^ The same strategy was applied for N-terminal modification of LHRH, which resulted in significant enhancement in the metabolic stability of the modified peptides in Caco-2 cell homogenate.^36^ The conjugation of lactose moiety to the N-terminal of [Gln^1^]-[d-Trp^6^]-LHRH led to a significant improvement in the absolute bioavailability of the peptide following oral administration to rats.^71^

Glycosylation improves the penetration of peptides across biological membranes.^72^,^73^ For example, glycosylation of endomorphin-1 resulted in a 700-fold increase in the membrane permeability across the Caco-2 cell monolayer, which could be due to transport through a lactose-selective transporter.^18^ N-terminal modification of LHRH with sugar moieties, including glucose, galactose and lactose, significantly improved its permeability.^36^ It was reported that GLUT2 and sodium–glucose linked transporter (SGLT1) contributed to the transport of the glycosylated LHRH analogues and the efflux pumps (P-gp and MRP2 transporters) only affected the apparent permeability the galactose derivative.^74^

## 3. Development of therapeutic peptides using glycosylation strategy

### 3.1. Neuropeptide therapeutics

Successful delivery of neuropeptides to the central nervous system for the treatment of neurological disorders has been hampered due to formidable obstacles, like the blood brain barrier (BBB), enzymatic digestion and liver clearance.^75^,^76^ Glycosylation has been shown to be an effective strategy to improve brain delivery of therapeutic peptides. This approach promotes the penetration of opioid peptides including enkephalins, endorphins and dynorphins into the brain and increases their pharmacological activity. The analgesic activity of the glycosylated opioid peptides including endomorphin-1 (through oral route) and enkephalin (intraperitoneal administration) has shown to be improved compared to the intact peptide and conventional analgesics, respectively.^18^,^64^ Enkephalin is a pentapeptide involved in antinociception with a short half-life in blood and an inability to pass the BBB. The attachment of Ser(Glc) residue to Leu-enkephalin amide (Tyr-d-Thr-Gly-Phe-Leu-Ser-NH_2_) improved the permeability of the opioid peptide across the BBB in mice. This glycosylated analogue produced a similar antinociceptive effect to morphine.^77^

**Fig. 9:**
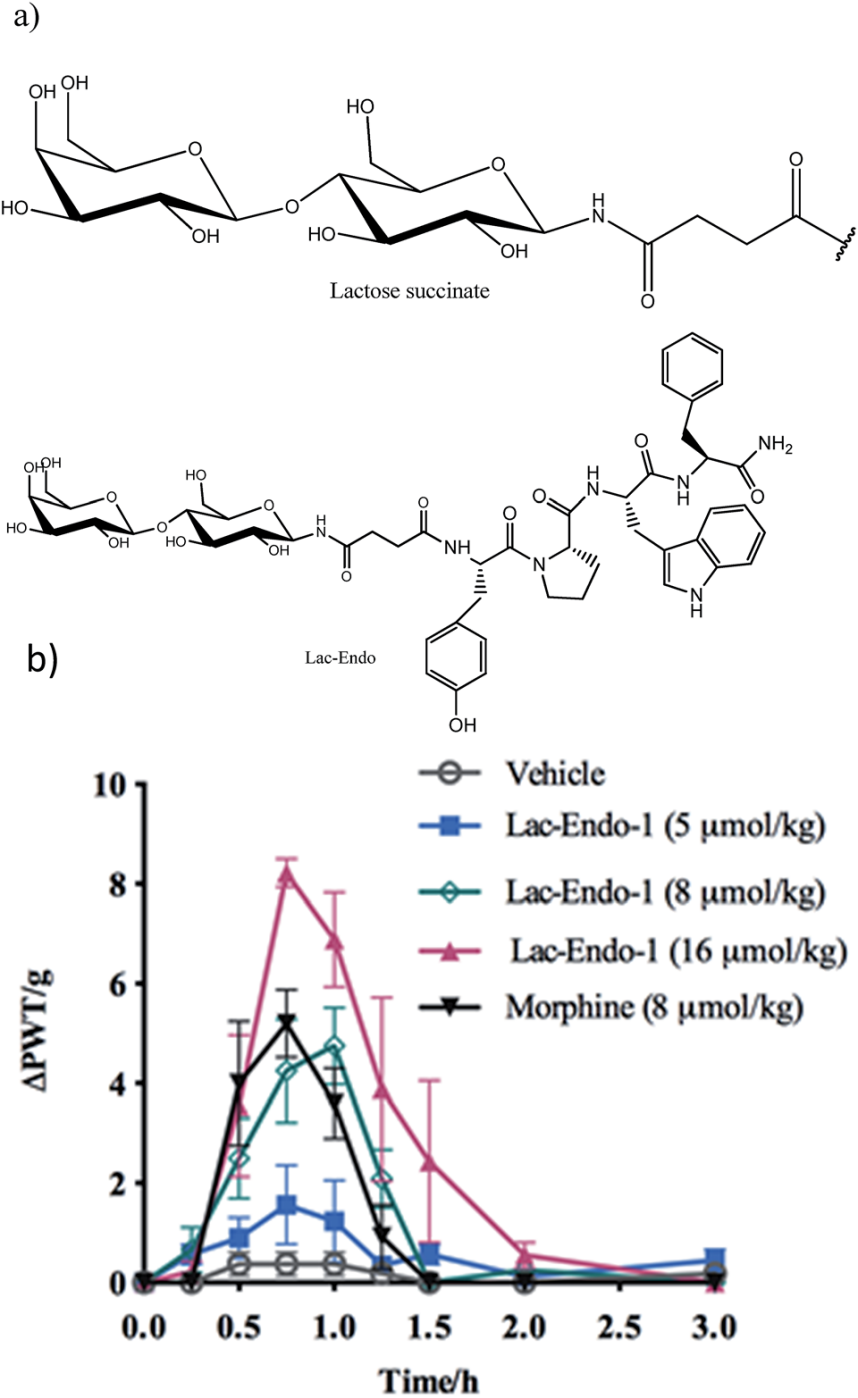
(a) Structure of the lactose (Lac)–succinate and Lac-endomorphin-1 and (b) time course of the antinociceptive bacterial effects of Lac-endomorphin-1, morphine, and vehicle in CCI-rats after oral administration. A single oral dose of Lac-endomorphin-1 produced dose-dependent analgesic activity in the ipsilateral hindpaws of a CCI-rat model of neuropathic pain.^18^ Lac-Endo-1, lactose-endomorphin-1; ΔPWT/g, normalised Δ paw withdrawal thresholds.

The improved permeability and higher metabolic stability of the glycosylated neuropeptides resulted in a significant increase in their bioavailability, which might account for the enhanced analgesic effect of the glycopeptides.^78^,^79^ The decreased renal clearance of the glycosylated analogue of Met-enkephalin (conjugated β-d-glucose) showed significant improvement in the bioavailability and analgesic effect of the peptide in rats.^80^ Conjugation of lactose succinamic acid to endomorphin-1 produced significant analgesic activity after oral administration in a chronic pain model of rats (Fig. 9).^18^ Polt *et al.* postulated that glycopeptides penetrate the BBB through adsorptive endocytosis;^81^ however, the exact mechanism is yet to be elucidated.

### 3.2. Radiopharmaceuticals

**Fig. 10:**
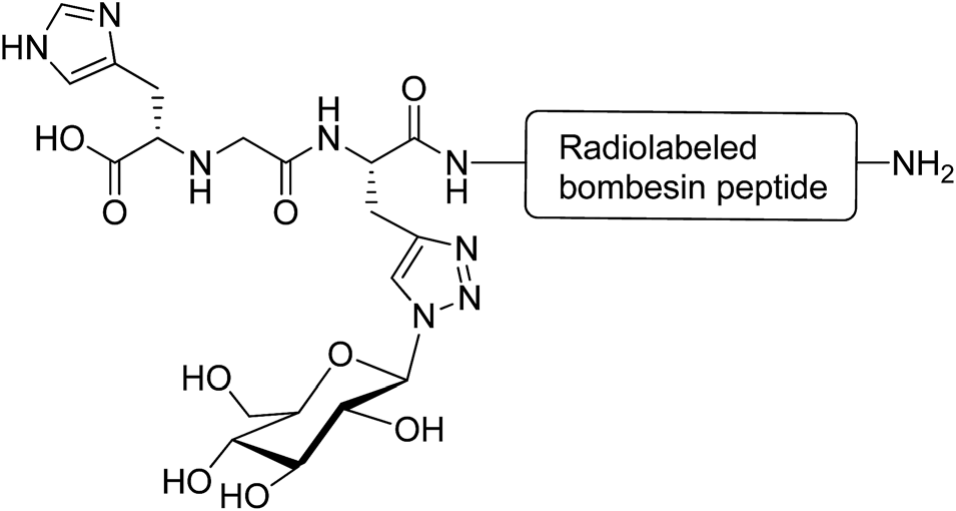
Glycated [^99m^Tc(CO)_3_]-labeled bombesin analogue. This compound was synthesised *via* a click reaction between azide derivative of glucose and a stabilised bombesin (7–14) sequence bearing the (N^α^His)Ac-chelator that modified with amino acid linkers containing propargylglycine residue.^84^

Glycosylation is a promising strategy for improving the biodistribution and poor pharmacokinetic profile of radiolabeled peptides for diagnostic and therapeutic purposes.^82^ The radiolabeled derivatives of the bombesin peptide have potential applications in cancer cell imaging and peptide receptor radiotherapy. However, they possess unfavourable pharmacokinetic properties, such as hepatic accumulation and hepatobiliary excretion.^83^ Conjugation of radiolabeled bombesin analogues with glucose moiety (through a triazole group) reduced abdominal accumulation and increased the uptake by tumours without affecting the cell internalisation of the modified peptides (Fig. 10).^84^ Glycosylation was applied to increase the hydrophilic property of radiolabeled Tyr(3)-octreotide peptide and overcome the drawbacks restricting its application in diagnostic imaging and cancer radiotherapy. Carbohydrate modifications of the peptide using glucose, maltose and maltotriose resulted in a higher renal clearance and subsequently less accumulation of the peptide in the liver and abdominal region. This modification made Tyr(3)-octreotide analogues (particularly maltose and glucose-conjugated peptides) suitable for targeted imaging and radiotherapy of somatostatin receptor-expressing tumours.^85^ In another study, the radiolabeled Arg-Gly-Asp (RGD) containing peptide (cyclysed pentapeptide Arg-Gly-Asp-d-Tyr-Lys) was glycosylated to improve the pharmacokinetic profiles of the modified analogues. It was observed that the conjugation of GlcNAc to Lys residue of the peptide decreased its lipophilicity and reduced the hepatic uptake, leading to a significant increase in tumour uptake. The improved biokinetic property of this glycosylated peptide made it a promising compound to be used for targeting tumours and angiogenesis imaging.^86^

### 3.3. Targeted delivery

Carbohydrate-mediated delivery, also termed glycotargeting, is a strategy that employs cell surface recognition in order to target specific organs. Carbohydrates are useful candidates for receptor-targeted peptide delivery as their receptors, known as lectin receptors, are expressed in the membrane of different cells, such as liver, tumour, and kidney cells. Therefore, the therapeutic agents conjugated with carbohydrate units can be recognised by those receptors and internalised into the cells.^87^ ASGPR is a lectin receptor expressed on the surface of liver hepatocytes that recognises the galactose and the galactosyl residue of the glycoproteins.^87^,^88^ ASGPR can be targeted for the delivery of peptides to the hepatocytes. Reports indicate that kidney and brain targeting is also achievable through glycotargeting.^89^–^92^ Glucose transporters (GLUT), such as GLUT1 and GLUT3, are overexpressed in various cancer cells, which can be targeted for anticancer therapy and immunodiagnostic markers.^89^–^92^ It has been found that the overexpression of GLUT1 is associated with tumour progression and the reduced expression of GLUT1 suppresses the tumour growth *in vitro* and *in vivo*.^91^,^93^,^94^

The impact of galactose, glucose and maltotriose on the pharmacokinetic properties of α-melanocyte-stimulating hormone was evaluated to target melanoma. It was shown that the glycosylated analogues exhibited excellent binding affinities (in nanomolar and subnanomolar ranges) to melanocortin receptor 1 that are overexpressed in melanoma cells *in vitro*. Among all glycopeptides, the analogue bearing galactose unit at the N-terminus of the α-melanocyte-stimulating hormone peptide had a favourable pharmacokinetic profile (higher tumour uptake with a lower kidney uptake) for melanoma targeting.^35^

## 4. Conclusions

The successful development of peptide-based therapeutics requires the optimisation of their pharmacological profiles. Glycosylation can be used to enhance the therapeutic behaviour of peptide drugs by optimising their pharmacokinetic properties. The incorporation of carbohydrate moieties into the sequence of peptides can change their physicochemical properties, leading to increased membrane permeability across biological membranes and improved proteolytic stability against digestive enzymes. The significant therapeutic potential of glycoconjugates accounted for the establishment of several techniques, which had important impacts on the development of carbohydrate-modified peptide drugs. Further understanding of the effect of glycosylation on the pharmacological properties of peptides is still required for the rational design of glycopeptides with enhanced biological activity.
